# A Practical Multi-Sensor Cooling Demand Estimation Approach Based on Visual, Indoor and Outdoor Information Sensing

**DOI:** 10.3390/s18113591

**Published:** 2018-10-23

**Authors:** Junqi Wang, Norman Chung Fai Tse, Tin Yan Poon, John Yau Chung Chan

**Affiliations:** Division of Building Science and Technology, City University of Hong Kong, Hong Kong, China; junqi.alan.wang@outlook.com (J.W.); typoon-c@my.cityu.edu.hk (T.Y.P.); yaucchan@cityu.edu.hk (J.Y.C.C.)

**Keywords:** HVAC, cooling demand estimation, occupancy density, vision-based occupancy detection, background subtraction

## Abstract

The operating efficiency of heating, ventilation and air conditioning (HVAC) system is critical for building energy performance. Demand-based control is an efficient HVAC operating strategy, which can provide an appropriate level of HVAC services based on the recognition of actual cooling “demand.” The cooling demand primarily relies on the accurate detection of occupancy. The current researches of demand-based HVAC control tend to detect the occupant count using cameras or other sensors, which often impose high computation and costs with limited real-life applications. Instead of detecting the occupant count, this paper proposes to detect the occupancy density. The occupancy density (estimated by image foreground moving pixels) together with the indoor and outdoor information (acquired from existing sensors) are used as inputs to an artificial neural network model for cooling demand estimation. Experiments have been implemented in a university design studio. Results show that, by adding the occupancy density, the cooling demand estimation error is greatly reduced by 67.4% and the R value is improved from 0.75 to 0.96. The proposed approach also features low-cost, computationally efficient, privacy-friendly and easily implementable. It shows good application potentials and can be readily incorporated into existing building management systems for improving energy efficiency.

## 1. Introduction

### 1.1. Background

The importance of improving the energy performance of buildings is widely acknowledged. The heating, ventilation and air conditioning (HVAC) systems are widely installed in large buildings to create a desired and comfortable indoor climate for occupants. As HVAC systems contribute a major proportion of building energy consumption (e.g., 54% of electricity is consumed by space cooling in office segment of Hong Kong [1] and 39.6% in commercial buildings of US [2]), reducing their energy consumption is of great importance. However, as the cooling demand varies irregularly over time, it is very difficult to maintain an optimal operation of HVAC systems [3,4]. Many research studies have revealed that a demand-based control is a powerful tool to facilitate an optimal performance of the HVAC system [4,5,6] and recent studies reveal that the demand-based control can lead to energy savings from 10% to 60% [7]. To realize such energy savings, it is necessary to determine the real-time cooling demand accurately and efficiently.

Basically, factors affecting the cooling demand can be categorized into several factors, namely external variables, internal variables and system characteristics [8]. System characteristics (including building location, orientation, building services systems, etc.) are normally fixed after building construction [9], while external and internal variables always vary during the operation. External variables are outdoor weather conditions (represented by outdoor temperature, humidity, solar radiation, wind speed, etc.), which can be readily obtained from the local weather station. Internal variables refer to the indoor objects generating or absorbing sensible or latent thermal load, which mainly involves occupants, lights and appliances. In commercial buildings, occupancy is a major random variable affecting the cooling demand. First, occupants produce heat, CO_2_ and contaminant. Second, the electrical appliances (radiators, fans, printers and other devices) and lighting systems, which produce heat, are also closely related to occupants’ activities [10]. Third, windows, doors and openings are mostly controlled by occupants, which affect the air leakage that brings heat exchange. These factors make the occupancy detection very important for an accurate cooling demand estimation [11,12,13]. With the accurate and sufficient occupancy information, the energy efficiency of HVAC systems can be significantly improved through better control and management as shown in Reference [14,15,16].

The occupancy information can be categorized into various scales, known as “occupancy resolutions”. In building sector, a popular definition of occupancy resolutions specifies three aspects, that is, space, occupants and time span [17]. The occupancy resolution varies from coarse levels to fine levels, for example, occupant presence, occupant count, occupant identity and activity type. A higher occupancy resolution level contains more information which enables better HVAC control and more flexible management. However, a higher occupancy resolution level also requires more advanced detection technologies or algorithms, which often means a higher cost with more complicated detection systems. Therefore, as claimed in Reference [18], it is important to determine an appropriate level of occupancy resolution based on the problem that needs to be solved, especially for practical applications with the limited budget.

Considering the random nature of occupancy and its importance to cooling demand, it is necessary to develop an effective and practical solution for occupancy detection with an appropriate occupancy resolution level. Next section will firstly review the existing occupancy detection technologies and identify the suitable ones. Then, the related work in HVAC regarding the identified detection technologies will be discussed. Finally, a suitable solution will be suggested for the real-time cooling demand estimation.

### 1.2. Related Work

#### 1.2.1. Occupancy Detection Technologies

The existing methods for occupancy detection can be broadly classified into direct and indirect approaches. The direct approach makes use of direct detection techniques, while the indirect approach relies on inferences. A great number of research works have been done on using the indirect inferences based on environmental information, such as CO_2_ concentration, temperature and relative humidity, which can be easily detected. However, indirect detection has many shortcomings. For the CO_2_-based detection method, the diversity of occupant activities, envelop infiltrations and delay of measurement are major causes of detection error. For instance, the CO_2_ generation rate is 0.27 L/min when a person is at rest and which becomes 0.53 L/min when a person is working. Thus, assuming a fixed CO_2_ generation rate will lead to a significant error in occupancy detection. Erroneous measurements also come from the envelop infiltrations. Temperature or relative humidity -based occupancy detection approaches are also suffered from time delays, diluted representation due to air mixing and low occupancy resolution [19].

The recent advances in information and communication technology enable the application of many direct detection approaches in occupancy detection [20,21], which generally have higher accuracy and reliability than indirect inferences. The direct detection approach can be realized through technologies like Wi-Fi [22], Bluetooth Low Energy (BLE) [19], RFID, Passive infrared (PIR) sensors [23] and so forth. It should be noted that PIR sensors are unable to detect stationary occupants [24]. For Wi-Fi, BLE and RFID, certain active devices (e.g., smart phone) or tags (e.g., RFID tag) should be powered and carried by occupants to establish an effective communication channel. In fact, the devices or tags are detected rather than the occupants, which pose some limitations on practical applications. First, these detection techniques completely depend on the devices or tags. To use these detection techniques, one needs to assume that every occupant carries the device or tag and turning on Wi-Fi or Bluetooth function all the time, which is not always the case. This also poses some inconvenience to occupants. Second, since these technologies (i.e., BLE and RFID) are not widely utilized in current building systems, additional investment is required to construct the infrastructure to enable these technologies. In addition, the concern of on the effect of radiated electromagnetic waves on human health poses some real hurdles on the use of these technologies, especially for the practical use of RFID technology in occupancy detection [24].

Table 1 summarizes the characteristics of various occupancy detection technologies. In summary, single sensor parameter or detection technology has its advantages and limitations. Considering that the indoor occupancy is highly chaotic and dynamic, utilizing a multi-sensor information seems to be a more promising option in achieving an accurate and reliable occupancy estimation, which can complement individual sensor limitations and reduce the risks of relaying on single data source. For instance, to improve the occupancy detection accuracy, CO_2_ concentration combined with relative humidity, temperature, air pressure, sound, lighting and PIR sensors are utilized in Reference [25,26,27,28]; Wi-Fi network and BLE network were used in Reference [19].

For practical applications, we need to consider not only the detection accuracy but also the implementation cost. As indicated in Table 1, the sensing technologies that are commonly installed, highly accurate and with low/no cost are Wi-Fi and vision-based detection. However, Wi-Fi requires devices to be carried and with Wi-Fi function turned on. Besides, signal stabilities and accuracy are major obstacles in practical applications [19]. Thus, the vision-based detection offers a more promising solution. As environmental sensors are commonly installed in building systems, the environmental data can be obtained easily and used as supplementary data. Thus, this paper tries to estimate the cooling demand by combining the environmental information (indoor and outdoor) and the visual information (from cameras).

#### 1.2.2. Occupancy Detection Using Cameras and Other Sensors for HVAC Applications

Research studies have been conducted on occupancy detection combining cameras and environmental sensors for HVAC applications. For example, Meyn et al. [29] adopted CO_2_ sensors, digital video cameras and PIR detectors for building-level occupancy estimation. Occupants in the video passing a line are counted (e.g., coming in or out). In Reference [30], an occupancy detection algorithm was developed to count the number of occupants crossing a virtual line near the room entrance. Wang et al. [31] proposed to use video and CO_2_ concentration for room occupancy estimation. The proposed video processing algorithm counts the occupant passing the entrance. These studies show a good detection accuracy by using cameras and environmental data.

However, the above vision-based occupancy detection algorithms have many difficulties in real practice. First, since these detection algorithms detect the objects passing the line, when multiple people walking across the line at the same time, significant errors may happen. The algorithm may also count loitering people several times. Second, these solutions require the cameras to be installed at special locations (e.g., at the entrance), which limits their applications since many existing cameras do not satisfy the requirements. Some studies used the object-based approach for video processing to achieve a high detection accuracy [32]. However, the computation of object (or feature) matching is exhaustive and powerful computing platforms are required, which means the cost could be high. Some studies also used 3D or depth cameras [33] for more accurate occupancy detection. However, these cameras are too expensive for practical applications. In addition to this, privacy concern is also a big problem. Yet there remains a challenge for developing a practical solution of indoor occupancy detection for the task of real-time cooling demand estimation. 

#### 1.2.3. Algorithms for Vision-Based Occupancy Detection

There are two usual vision-based approaches for estimating occupancy: pixel-based approach and object-based approach [35]. Pixel-based methods detect pixels from a moving object, where a fixed background is assumed. This method generally works well for indoor scenarios with a fairly static background. However, it is not as reliable in most outdoor scenes due to more dynamics involved like wind and illumination. Current researches focus on modeling of the pixels in the background and updating of such models [35]. On the other hand, object-based methods detect specific objects using the features in the foreground. Human body features (like face, head, shoulder) can be constructed for detection. In controlled scenarios (e.g., indoor), pixel-based methods work well and generally require a reasonable computation [35]. The object-based methods can handle complicated scenes (e.g., outdoor) but are computationally exhaustive requiring high-resolution cameras and powerful computing platforms. Thus, the pixel-based approach offers a better solution to achieve a fast, low-cost and real-time image processing for indoor environments.

In pixel-based methods, background subtraction (also known as foreground detection) is a widely used approach for detecting moving objects in videos obtained from stationary cameras. The major application field of background subtraction is in surveillance [36]. Basically, background subtraction differentiates the current frame and a reference frame (or “background model”). Different background subtraction algorithms can be found in literatures [36] and one of the most popular background subtraction algorithms is the Gaussian mixture model (GMM) proposed by Stauffer and Grimson [37]. This technique assumes that each pixel in the video can be represented by a mixture of Gaussian distributions (a sum of weighted Gaussians) which can be further updated in an online manner. Although the GMM performs well, the change in illumination and the coming in and out of objects in the scene still present real challenges.

To tackle these challenges, many improvements have proposed in GMM-based background subtraction approaches. Kaewtrakulpong and Bowden [38] modified the updating equations in Reference [37], which leads to a faster and more accurate adaptation to the changing illumination. Chen et al. [39] considered combining the pixel-based and block-based approaches in a hierarchical structure, which enables the identification of non-stationary background and the detection of rough foreground objects. Among these improved GMM-based algorithms, a popular one is the adaptive GMM proposed by Zivkovic [40,41]. This adaptive GMM can automatically compute the required number of Gaussian components on-line, which allows good adaptation to the varying scene (like varying illumination). Meanwhile the processing time can be reduced and the segmentation can be enhanced. The implementation is also simple and computationally friendly.

### 1.3. Summary

In summary, to achieve an accurate cooling demand estimation with an affordable cost, an appropriate level of occupancy information is vital. Combining environmental sensors and cameras offers a promising solution. However, the current solutions in HVAC fields tend to detect the occupant count, which often impose high computation complexity and high costs with limited real-life applications. Since the cooling demand estimation does not require an accurate number of occupants, an estimation of the occupancy density will be more appropriate, efficient and cost-effective. Nevertheless, using the information of occupancy density for load estimation has not been well investigated. Thus, this study proposes a pixel-based approach to capture the foreground moving pixels for representing the occupancy density. With the pixel-based approach, cameras at the surveillance-level can be employed, resulting in an affordable solution. The requirement of computation power is also low and a micro controller unit (MCU) can be used to process the video internally, which minimizes the privacy concern with a further reduction in cost.

## 2. Methodology

### 2.1. Overview

**Figure 1:**
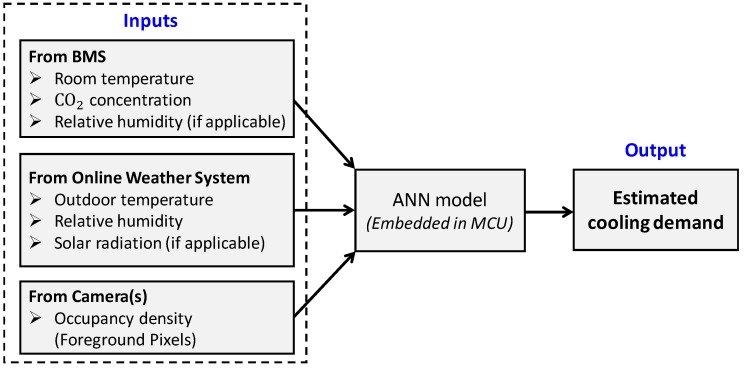
Block diagram of the proposed load estimation scheme.

An ANN-based algorithm is proposed to build the cooling demand estimation model (heating is not considered here) of an indoor space because the ANN has been successfully used in predicting occupancy and demand for demand-based HVAC control [10,42,43,44]. In Figure 1, the input parameters gathered by a MCU include room environmental conditions (from the building management system (BMS)), outdoor weather conditions (from an online weather system) and occupancy density (from camera), which are used as the inputs to the ANN model.

1. Room environmental conditions may include room temperature, relative humidity and CO_2_ concentration, which are gathered by the BMS.
2. Weather conditions may include outdoor temperature, relative humidity and solar radiation (if there are external walls or windows), which can be obtained from an online weather system.
3. Occupancy density is estimated by the number of foreground moving pixels based on the background-subtracted images.

The specifications of the camera used for the occupancy estimation in this study:

(1) should cover the whole, or most, of the occupied space;
(2) does not pan and tilt;
(3) does not have fisheye lens;
(4) does not have any automatic control, such as, zooming, exposure and white balance.
(5) the resolution of the used cameras is close to the building surveillance camera with a usual resolution of about 320 × 240 pixels (thus, the proposed approach can also make use of existing surveillance camera systems if the privacy issue is properly solved).

### 2.2. Occupancy Density Estimation Based on Foreground Moving Pixels

An adaptive Gaussian mixture model (GMM) [40,41] is adopted in this study to extract the foreground pixels (which are moving pixels) from a surveillance-level video image. The occupancy density is represented by the density of foreground moving pixels (DFMP) rather than the precise occupant counts, which greatly reduces the computation complexity. The background subtraction is firstly conducted to obtain the foreground image which mainly consists of the moving occupants. Next, the occupancy density is estimated by the ratio of moving pixels to total image pixels (see Equation (1)).

(1) $$Occup_{Density}\approx \mathrm{DFMP}=\frac{Num_{pix,moving}}{Num_{pix,tot}}$$

where *Occup_Density_* is the occupancy density, *Num* is number, *pix* is pixel, *tot* is total.

For the purpose of cooling demand estimation, using the DFMP to represent the occupancy density in the occupied space is adequate and suitable. The reasons are that: (1) DFMP increases as the number of occupants increases assuming that persons are of equal-size [45]; and (2) the DFMP increases as the occupant’s activity level (or metabolic rate) increases. Therefore, the DFMP not only represents the occupant density but also reflects occupants’ activity level. 

Take walking and seating as an example. When an occupant is walking, the whole body is moving and is counted as moving pixels. When an occupant is seating, only the upper body may be moving and is counted as the moving pixels. Thus, walking generally produces more moving pixels than seating, which agrees with the fact that walking generally has a higher activity level than seating. Different activity levels have different heat generation rates, resulting in different cooling demand. In Table 2, metabolic rates at different activities are shown based on the data from ASHRAE handbook [46] and the general relationship with the DFMP is also presented. 

### 2.3. Adaptive GMM

The adaptive GMM algorithm updates both the parameters and number of Gaussian components for each pixel. It is able adapt to the change of background settings (e.g., furniture) in the occupied indoor space so that human intervention is minimized. Another advantage is the ability in adapting to sudden change in lighting level. For instance, occupants may turn off part of the lights when viewing video and presentation and so forth. Such changes in lighting environment would cause a spike of subtracted foreground pixels, which can be easily mitigated by the adaptive GMM. 

The adaptive GMM is introduced briefly as follows. Equation (2) is used to decide if a pixel belongs to the foreground (*FG*) or background (*BG*) using the value of D.

(2) $$D=\frac{p(BG|x_{\left(t\right)})}{p(FG|x_{\left(t\right)})}=\frac{p(x_{\left(t\right)}|BG)p\left(BG\right)}{p(x_{\left(t\right)}|FG)p\left(FG\right)}$$

where $x_{\left(t\right)}$ is the value of a pixel at time t in a certain color space (e.g., RGB).

Since we do not have information on the *FG* objects, a uniform distribution is assumed for *FG* objects, that is, $p\left(x_{\left(t\right)}|FG\right)=c$. The *BG* model is estimated from a training set ℵ, which is represented as p(x|ℵ,BG). For adapting to background changes, a time interval T is used and the training set is ${ℵ}_{T}=\left\{x_{\left(t\right)},\ldots ,x_{\left(t-T\right)}\right\}$. ${ℵ}_{T}$ is updated for every new sample. As the sample may contain *FG*, the density is estimated by GMM with M components:

(3) $$p\left(x|{ℵ}_{T},BG+FG\right)=\overset{M}{\underset{m=1}{\sum }}{\hat{\pi }}_{m}N\left(x;{\hat{\mu }}_{m},{\hat{\sigma }}_{m}{}^{2}I\right)$$

where ${\hat{\mu }}_{m}$ is the estimate of mean and ${\hat{\sigma }}_{m}{}^{2}$ is the estimate of variance of a Gaussian component, I is the identity matrix, ${\hat{\pi }}_{m}$ is the estimated mixing weights (${\hat{\pi }}_{m}\geq 0,\sum {\hat{\pi }}_{m}=1$). 

There are three steps of adaptive GMM algorithm:

(1) Classify the new sample $x_{\left(t\right)}$ with: $p\left(x_{\left(t\right)}|{ℵ}_{T},BG\right)>\delta$
(2) Update: $p\left(x|{ℵ}_{T},BG+FG\right)$
(3) Update: $p\left(x|{ℵ}_{T},BG\right)$

(δ is a threshold for deciding if $x_{\left(t\right)}$ belongs to *BG*.)

**Figure 2:**
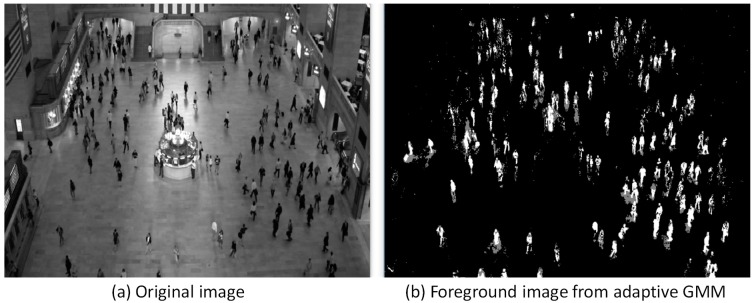
Images before and after processing (using adaptive GMM) [47].

Noted that the detailed updating equations and Gaussian component number selection can be found in Reference [40,41] and are not repeated here. Python or Matlab can be used to implement the adaptive GMM algorithm. This study adopts the function “BackgroundSubtractorMOG2” in OpenCV (Open Source Computer Vision Library, version 3.3.0) by using Python. Example images before and after processing are shown in Figure 2, where a typical sample video taken at Grand Central Station in New York (downloaded in Reference [47]) was used for demonstration. The adaptive GMM transfers the original image into gray scale (0–255). The white color (or 255) is defined as *FG*, while the black color (or 0) is used for *BG*. The adaptive GMM can also detect shadows which are marked as gray color (values in between 0 and 255). 

### 2.4. ANN Modelling

The ANN model consists of an input layer, a hidden layer and an output layer. An adaptive Levenberg-marquardt algorithm is used to train the relationship between the input variables and the measured cooling load of the occupied space [48]. The root mean squared error (RMSE) and Pearson correlation coefficient (R) are used for the model evaluation. Matlab is used for ANN model training and implementation.

## 3. Experiment Setup

### 3.1. Test Room and Cooling System

**Figure 3:**
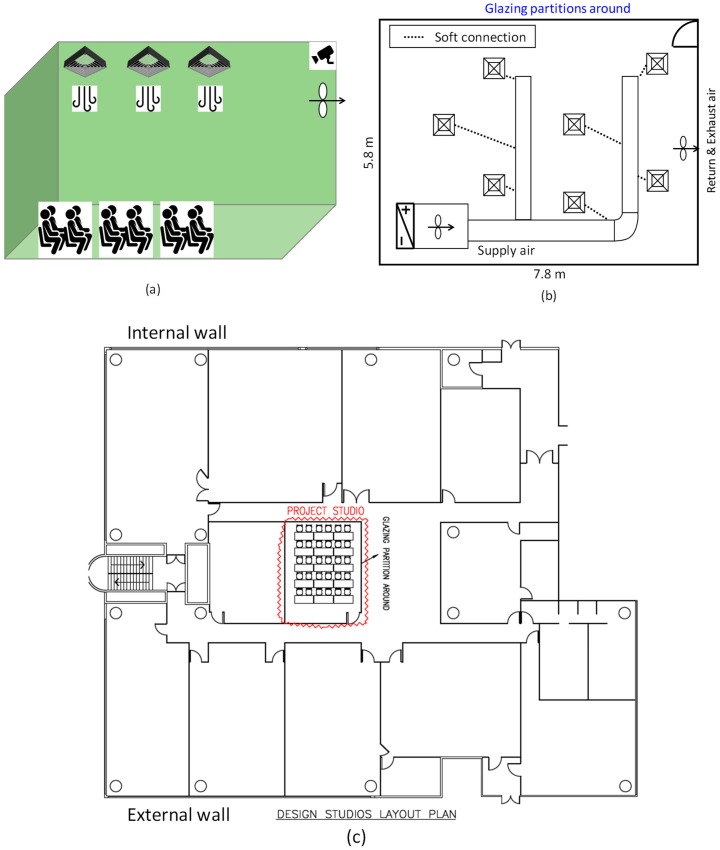
Test room: (**a**) 3D visualization; (**b**) cooling system schematic; (**c**) design studios layout plan.

The experiment was taken place in a project studio in a university in Hong Kong. An information summary of the project studio is shown in Table 3. The project studio has an area of 45 m^2^ that can accommodate a maximum of 30 students, the room and cooling system schematics of which are shown in Figure 3. There is one projector, one PC and eight luminaries, which are turned on when occupied. The project studio locates at the interior zone (as shown in Figure 3c), which does not have the external windows or daylighting. Thus, for simplicity, the external solar radiation is not considered as one of the input variables in the cooling demand estimation.

The camera was installed at the top corner of the room (see Figure 3a) to minimize the object occlusion for the detected area. There is a constant air volume (CAV) system consisting of 7 air diffusers for conditioning the room air, which supplies an air volume of 900 L/s (see Figure 3b). The outdoor air is supplied separately to the room. A proportional-integral (PI) algorithm is adopted to modulate the water valve based on the difference between the return air temperature and the room air temperature set point. The room air temperature is fixed at 22 °C while the room humidity is not precisely controlled.

### 3.2. Sensing System and ANN Algorithm

**Figure 4:**
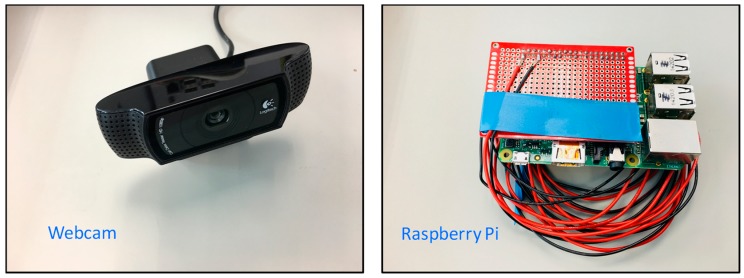
The webcam (**left**) and Raspberry Pi (**right**).

Table 4 summaries the data collected in the experiment. There are CO_2_ sensors and temperature sensors located in the return air duct and the supply air duct respectively. The data from these sensors are collected from the central BMS. The weather condition, including temperature and relative humidity, is acquired from an online weather system [49]. The update interval of the system is 30 min. Instead of using a real surveillance camera, a webcam was used as the substitution of surveillance camera in the experiment setup (see Figure 4), which is installed at the room ceiling (see Figure 3a). The resolution of webcam was set to 320 × 240 to resemble the resolution of the surveillance cameras used in the university campus. 

The heat gains from lighting and equipment can be represented by the DFMP for the following reasons. When the room is occupied, that is, DFMP is larger than “0”, lighting and equipment are all turned on and the corresponding heat generation can be considered as a constant value. When the room is not occupied, that is, DFMP is “0”, lighting and equipment are all turned off and the heat generation is zero. Thus, the heat gains from lighting and equipment are either “0” or a constant value and its relationship with the DFMP can be learnt by the ANN model. 

**Figure 5:**
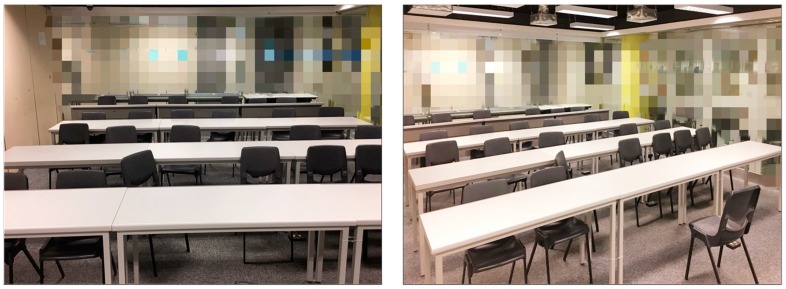
Screenshots of the test room.

The MCU, Raspberry Pi [50], was used to gather and process the information (see Figure 4). The video image from the camera was firstly background subtracted to remove the stationary background. Two screenshots of the test room are shown in Figure 5. The project studio has glazing partitions but curtains were pulled down during occupied periods to avoid exterior interruptions. The video data from the camera was directly and automatically processed in Raspberry Pi without the need of human manipulations, which minimizes the privacy concerns. During the experiment, no restrictions were imposed on occupants regarding the use of the design studio to resemble the near real-life operations. 

All the data was synchronized and processed by the ANN algorithm to learn the relationship between the cooling load and the input parameters. A single layer network with 10 neurons in the hidden layer was used in the experiment.

The reasons are that: (1) it produced a satisfactory accuracy and the increased in the number of neurons did not make a significant improvement; (2) overfitting is always encountered when a large number of hidden neurons is used [51]. Therefore, 10 hidden neurons were used to avoid the use of unnecessarily complex functions. Consequently, 70% of the data was used for training, 15% for validation and 15% for testing. 

### 3.3. Test Days and Weather Data

**Figure 6:**
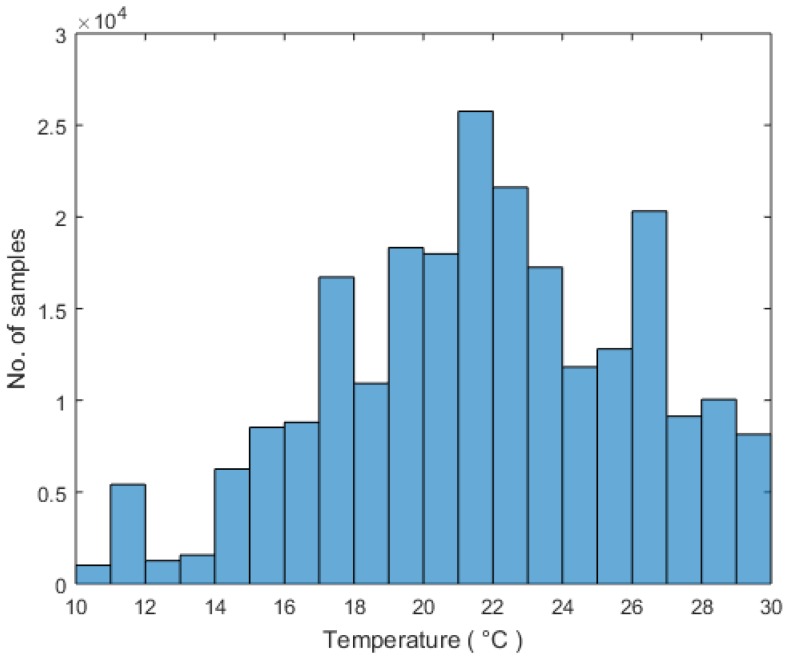
Histogram of the outdoor temperature.

30-day data was gathered from 09:00 to 21:59 across four months in the year of 2016, during which a wide variation in the weather condition was observed. The dates of experiment and the summary of the weather statistics are shown in Table 5. During the experiment, the temperature ranged from 10 to 30 °C and the relative humidity varied from 25 to 100%. A maximum temperature difference of 15 °C was observed on a single day and the maximum relative humidity difference was 63%. Figure 6 shows the histogram of the outdoor temperature. 

## 4. Results

### 4.1. Relationships between DFMP and Activity Type

In Section 2.2, it is claimed that DFMP increases as the occupant’s activity level (or metabolic rate) increases. This part will show the experimental results to illustrate this assumption. It should be noted that the detection logic only counts the white color pixels (equal to 255) and gray color pixels are not counted to prevent the influence of shadows. This experiment was taken at a classroom (not the project studio) with daylighting to test the adaptive GMM in a contrasting background. 

**Figure 7:**
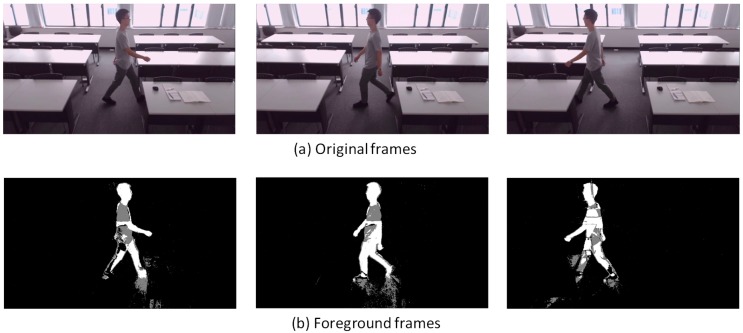
Walking (1.8 m/s)—sample images.

**Figure 8:**
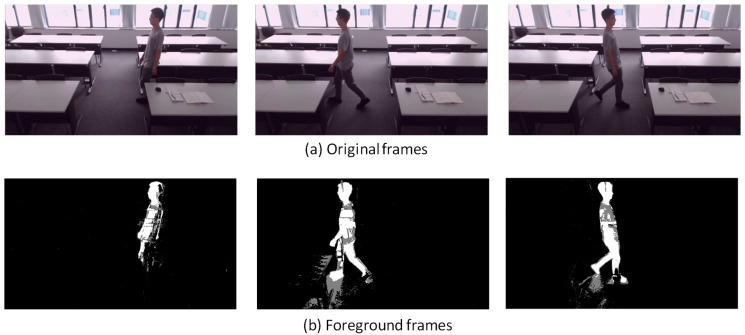
Walking (1.2 m/s)—sample images.

**Figure 9:**
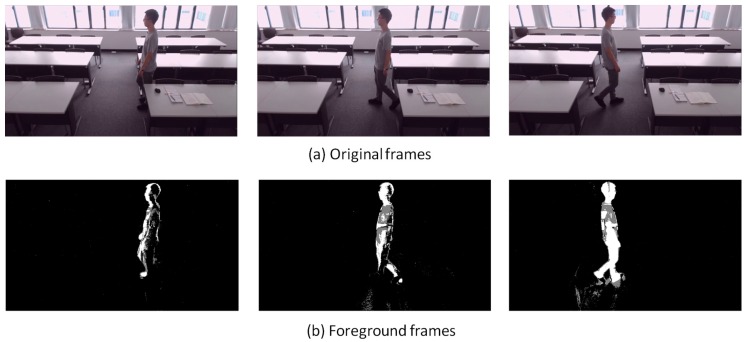
Walking (0.9 m/s)—sample images.

**Figure 10:**
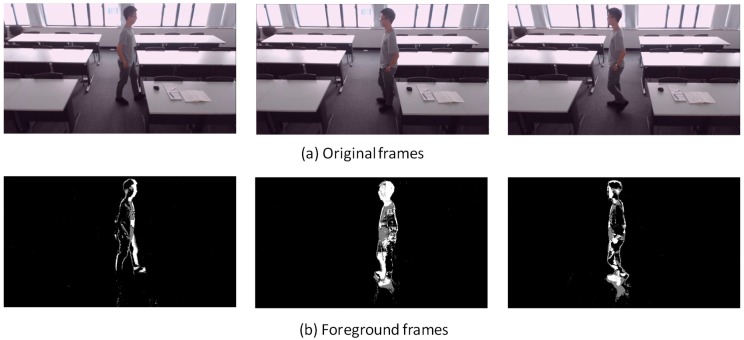
Walking about—sample images.

**Figure 11:**
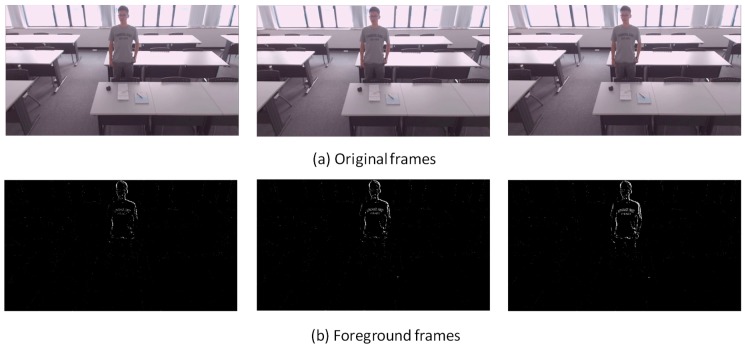
Standing relaxed—sample image.

**Figure 12:**
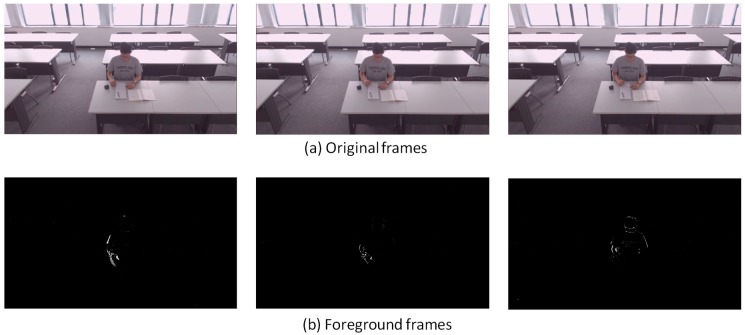
Reading and/or writing—sample images.

**Figure 13:**
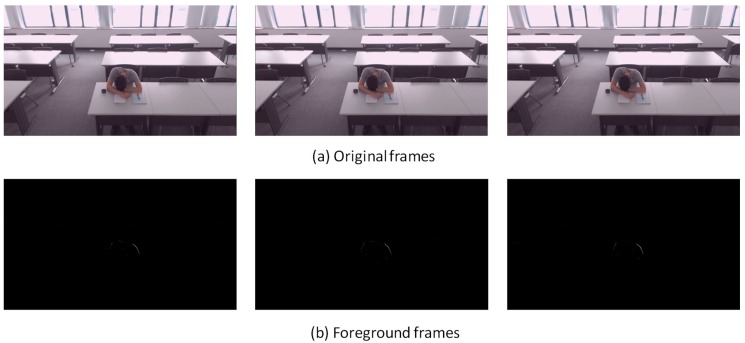
Sleeping—sample images.

Figure 7, Figure 8, Figure 9, Figure 10, Figure 11, Figure 12 and Figure 13 show the original frames and processed foreground frames of different activity types, including walking, standing, reading/writing and sleeping. The adaptive GMM captures the moving pixels in terms of a stationary background. Basically, the more intensive the moving activity is, the more moving pixels will be captured. In terms of the walking activity, more moving pixels will be captured with higher walking speeds. For example, Figure 7 captures almost the whole moving human body in foreground frames, while Figure 9 and Figure 10 captures part of the human body. For standing, reading/writing and sleeping (See Figure 11, Figure 12 and Figure 13), the captured moving pixels are fewer since the moving of human body is not significant. For instance, there is nearly no moving pixels captured in sleeping status (Figure 13) as the entire human body is almost stationary.

**Figure 14:**
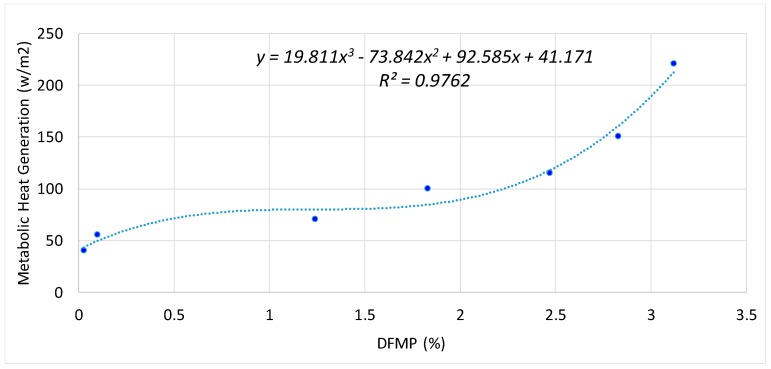
Metabolic Heat Generation Rate vs. DFMP.

Table 6 shows the Metabolic Heat Generation (w/m^2^) (acquired from ASHRAE handbook [46]) and detected DFMP (%) using Adaptive GMM of the tested office activity types. Figure 14 shows the plot of Metabolic Heat Generation (w/m^2^) and the detected DFMP (%). The relationship between Metabolic Heat Generation and detected DFMP is not a linear relationship but can be regressed by a cubic function, *y* = 19.811 × 3 − 73.842 × 2 + 92.585*x* + 41.171, with a *R*^2^ of 0.9762, which shows a good prediction accuracy. This validates the assumption that the occupant’s activity level (or metabolic rate) is proportional to the proposed DFMP. 

It should be noted that the selected activity types are well-defined activity types from ASHRAE handbook [46]. The activity types that can be hardly defined (e.g., heavy machine work) were not selected. Metabolic Heat Generation Rates (w/m^2^) were estimated based on the activity types using the ASHRAE table [46]. The developed cubic function of Metabolic Heat Generation Rate is only to show the form of the relationship and is not used in the cooling demand estimation model since ANN can learn the relationship between the DFMP and the cooling demand directly.

### 4.2. Cooling Demand Estimation

This section presents the results of the cooling demand estimation with vison (by the proposed algorithm), as compared to the actual measured cooling load and the estimated cooling demand without vision. The calculation of the actual measured cooling load is as follows. As a single CAV AHU is installed in the design studio, the air flow can be assumed constant. Therefore, the amount of cooling supplied by the AHU can be calculated by Equation (4) (which was also adopted in Reference [52]). The measured data of supply and return air temperature was used. Only the sensible part of the space cooling load is calculated in this study due to the lack of humidity sensors for supply and return air ducts. For simplicity, the ventilation load for conditioning outdoor air is not computed. We used the sensible space cooling load to demonstrate the proposed method.

(4) $$Q_{sens}\left(i\right)=\rho \times v_{air}\times c\times \left[t_{r}\left(i\right)-t_{s}\left(i\right)\right]$$

where

$t_{r}$ is the return air temperature (°C)
$t_{s}$ is the supply air temperature (°C)
ρ is the air density (kg/m^3^)
$v_{air}$ is the air volume flow rate (m^3^/s)
c is the specific heat capacity of air [J/(kg·°C)]
$Q_{sens}$ is the sensible space cooling load (W)
i is the time index

Since the data from various sources is captured at different time intervals, they were firstly synchronized by simple interpolations. There were 7801 samples collected in a typical day from 9:00 to 21:59, with a 6-s time interval between two consecutive samples. Due to the scheduled operation of the central cooling system, when the cooling system is shut down, the supply air temperature will be slightly higher than the return air temperature due to the heat gain in return and supply air ducts, resulting in a negative value of calculated cooling load. Thus, this portion (around 5%) of the data would not be used in the study.

**Figure 15:**
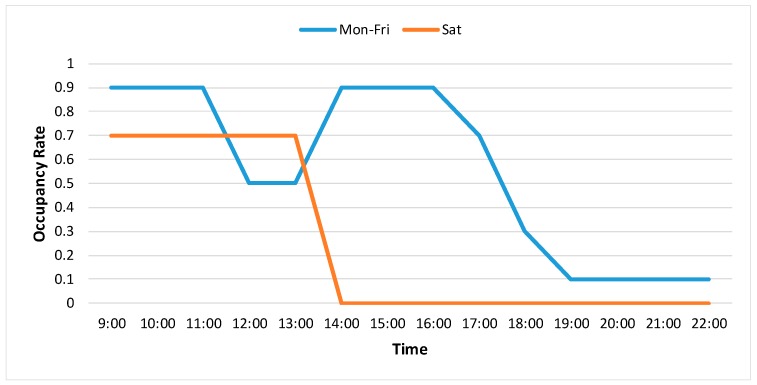
Occupancy Schedule.

For the cooling demand “without vision”, a typical occupancy schedule was assumed (see Figure 15) and the input information contains room temperature, room CO_2_ concentration, outdoor weather condition and the designed occupancy schedule.

**Figure 16:**
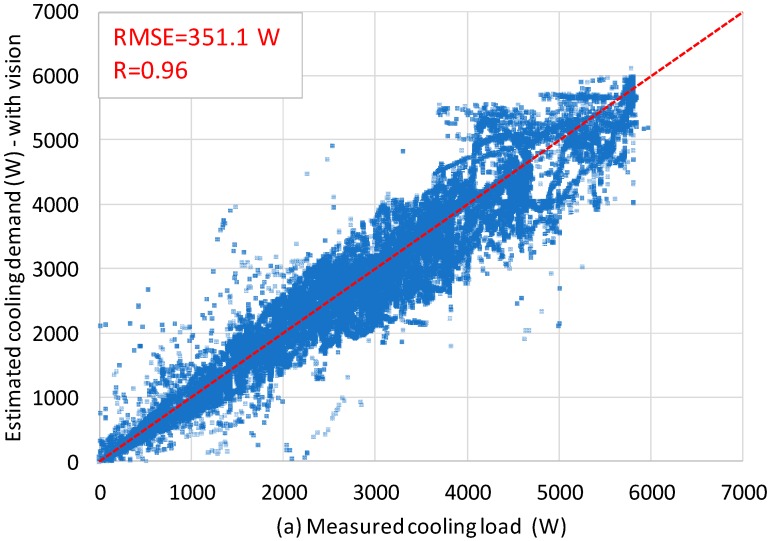

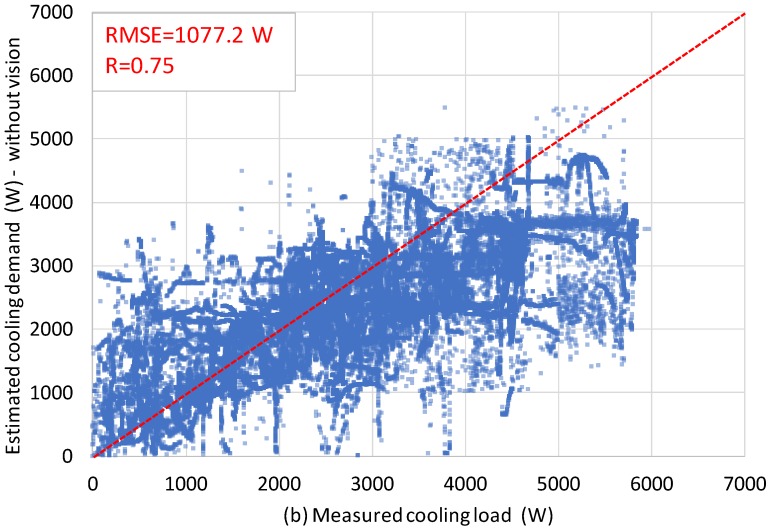
Measured and estimated cooling demand (**a**) with vision-based occupancy information; (**b**) without vision-based occupancy information (darker area represents more data points).

Figure 16 shows the measured cooling load against the estimated cooling demand, with and without vision-based occupancy information respectively, where the dotted red line represents the perfect estimation. For the entire experiment period (29 February 2016 to 2 May 2016), the RMSE of the cooling demand estimation with the vision-based occupancy information was 351.1 (Watt), as compared to 1077.2 (Watt) without the vision-based occupancy information. In other words, the RMSE was reduced by 67.4% when the vision-based occupancy information was added. In terms of the correlation coefficient (R), a value of 0.96 was achieved with the vision-based occupancy information as shown in Figure 16, which shows a significant positive linear relationship between the measured and the estimated cooling demand. Without the vision-based occupancy information, the correlation coefficient is only 0.75.

**Figure 17:**
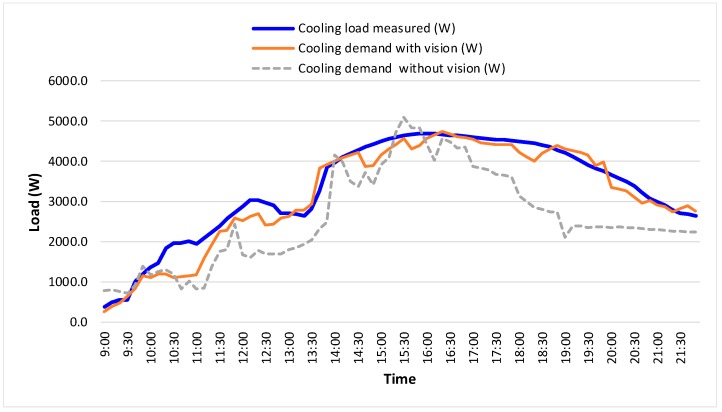
Instantaneous load in one day.

Figure 17 shows the instantaneous values (sampled in 10 min interval) of the measured cooling load and the cooling loads with & without vision in one day. As can be seen, for the measured cooling load, it raises gradually at first. Then, it has a drop during the noon break (around 12:00–13:30). The maximal load appears in afternoon (around 15:30–16:00). After that, the cooling load decreases. This depicts a typical cooling load profile of the test room. 

For the estimated cooling load “with vision”, the DFMP is used as one of the input variables. For the estimated cooling load “without vision”, a designed typical occupancy schedule is used as one of the input variables. The other input variables are the same, that is, environmental variables and outdoor weather condition. 

From Figure 17, a big difference can be observed between the estimated cooling load (“without vision”) and the measured cooling load. The main reason is that the actual occupancy condition is more chaotic and could be different from the designed occupancy schedule. In contrast, the estimated cooling load (“with vision”) follows the measured cooling load more closely since the detected occupancy density is used.

## 5. Discussions

### 5.1. Cooling Demand Estimation Performance and Implications

This paper firstly investigates the use of density of foreground moving pixels (DFMP) acquired from visual information in the cooling demand estimation. The effectiveness of using the DFMP to estimate the occupancy (or crowd) density has been demonstrated in previous researches [53]. However, those studies mostly focused on the applications in surveillance, crowd management and security. For the HVAC research, it has not been done to correlate the DFMP with the human metabolic heat generation rate. Results demonstrate that the proposed DFMP can predict the single human metabolic heat generation rate by a cubic function with a *R*^2^ of 0.9762.

As shown in Figure 16, by incorporating the vision-based occupancy density information (or DFMP) into the cooling load estimation, the *R* value of estimation was greatly enhanced from 0.75 to 0.96 and the RMSE was reduced by 67.4%. The probable reason is that the proposed DFMP contains information of not only “occupant count” but also “human metabolic heat generation rate”. Besides, the DFMP is also related to the operation of any occupant-related equipment (e.g., lighting and PC). All these factors contribute to the amount of cooling demand, which makes DFMP suitable for cooling demand estimation. 

The uncertainty of the measured variables will bring errors into the cooling load calculation. However, as the three load values (i.e., the measured load, the estimated load without vision and the estimated load with vision) were based on the same experimental data, they would have the same measurement uncertainty. Thus, the measurement uncertainty will not prejudice the improvement made by the proposed method, though the measurement uncertainty needs to be considered when calculated cooling load is to be compared with the actual cooling load.

### 5.2. Cost and Computation

In recent years, object detection is growing rapidly. Some object detection algorithms can achieve a high detection accuracy, for example, Faster RCNN [54] and YOLO [55]. However, the computation of the deep neural network still requires a NVidia Pascal Titan X GPU (1800 UD$) to achieve a 30-fps real-time processing speed [56]. Such a GPU is too expensive for a low-cost sensing solution. Besides, object detection algorithms output the object count, which is not necessary for cooling demand estimation. Thus, this study did not adopt the object detection algorithms.

As the indoor and outdoor environmental information can be obtained from the existing sensors or online weather station, no additional cost is incurred. The video camera adopted in the experiment is at surveillance level, which costs only around US$ 40–80 only. The MCU, raspberry Pi, costs around US$ 40–50. Compared with the solutions using depth or 3D camera and powerful desktop PCs (costing thousands of US$), the proposed pixel-based occupancy estimation approach offers a very economical solution. The computation of the proposed solution is fast (quicker than the sampling time) and can satisfy the requirement in real-time applications. 

### 5.3. Potential Applications

There are several potential applications of the proposed cooling demand estimation approach. First, since the presence of occupants is random, the proposed approach can detect whether the room/zone is occupied or not. With this information, when room is not occupied, energy consumption can be greatly reduced by resetting the indoor temperature set-point or switching the cooling devices to standby-mode as discussed in Reference [12]. The vision-based occupancy detection scheme has advantages over CO_2_-based, Wi-Fi based, RFID and PIR as discussed in the introduction of this paper. 

Another potential use is to integrate the proposed algorithm with the conventional temperature-based proportional–integral (PI) control that is widely used in HVAC systems. The temperature sensor is usually installed on the wall or inside the return air duct, which is not the real temperature of human activity regions. A major problem of temperature-based PI control is that it suffers from slow response and time delay [57], which may result in inefficient HVAC operations and poor thermal comfort [58]. The proposed real-time cooling demand estimation approach can be used to improve the conventional PI control of HVAC systems.

Moreover, the visual information can be used to estimate the spatial distribution of cooling demand of large-scale spaces. Such information will enable a better and more flexible local-level management of indoor environment. For instance, in a large-scale space such as a lecture theater or conference room, people may scatter randomly in the space, which results in a non-uniform cooling demand distribution leading to over or insufficient cooling [58]. The proposed cooling demand estimation approach can help to allocate the cooling/fresh air output of each equipment in real-time, hence the local cooling demand can be met in a proper way and energy efficiency can be improved [59,60,61]. 

### 5.4. Limitations

While a close relationship between the DFMP and the occupant density has been widely recognized, the relationship between the DFMP and occupants’ activity level has rarely been pointed out before. This paper conducted a simple experiment to demonstrate the relationship between the DFMP and occupants’ activity level (see Section 2.2) for the case of single person. The experimental verification involving multiple person should be done in future. As the “cubic” relationship was not used in the cooling demand estimation model, it will not affect the result. Besides, this paper only estimated the metabolic rate. The measurement of metabolic rate (representing the human activity level) is the topic of Physiology or Clinical Nutrition, requiring complex calculations and measurement devices and so is out of scope of the present paper and could be done in the future.

The experiments did not measure the relative humidity due to the lack of humidity sensors. Thus, only sensible space cooling load was used to verify the proposed load estimation method. After installing humidity sensors, it is still convenient to use enthalpy for the load calculation. This can be done in future. Since the amount of the latent cooling load depends on the occupancy density and degree of activity, it can be expected that the proposed DFMP can also improve the estimation accuracy of the latent cooling load, as compared to the use of a pre-determined occupancy schedule. Thus, the general conclusion drawn from the results based on the sensible space cooling load is still meaningful and will not be affected drastically by using enthalpies or specific heat and temperatures. It should be stressed that the proposed method can work for both sensible and total cooling loads.

The experiment was not taken place in consecutive weeks because the communication network is not secure during the experiment period. However, the experiment contained 30-day data across four months and the covered weather conditions (shown in Table 5) can reflect typical local weather conditions and hence are sufficient for the performance verification. As the proposed approach requires the inputs of real time environmental parameters obtained through internet, a secure and reliable communication link is to be ensured to maintain a reliable control. 

The proposed cooling demand estimation algorithm can be deployed on a low-cost compact MCU, which warrants an easy installation in an affordable budget. Nevertheless, further research work will focus on the infrastructural requirement for a mass deployment, such as the configuration of the system architecture, the required network bandwidth for extra data collection for the distributed controllers and so forth. Privacy is also a big concern for any vision-based detection solutions. This study uses an embedded MCU which processes the video internally and only outputs the occupancy density without recording. The internal processing brings the threat on privacy to a minimal level. However, to further improve the video data security, the data can nevertheless be encrypted in the MCU [62].

## 6. Conclusions

The estimation of cooling demand is very important for building energy consumption and management and occupancy is a surely primary random influencing factor in the cooling demand. This paper studies the real-time cooling demand estimation based on multi-sensor data involving visual, indoor and outdoor environmental information. Instead of detecting the occupant count, this study detects the occupancy density for estimating the cooling demand. The pixel-based method (i.e., adaptive GMM) is used to extract the foreground moving pixels as a representation of occupancy density. Experimental results show that, the inclusion of the vision-based occupancy density information improves the R value from 0.75 to 0.96 and reduces the RMSE by 67.4%. It is demonstrated that the use of the proposed approach in representing occupancy density is very suitable for cooling demand estimation as it represents both the occupants’ number and activity levels (or metabolic rate) that contribute to the cooling demand. 

The proposed sensing approach also has several practical values: (1) surveillance-level cameras can be used, which saves cost and improves the applicability; (2) since the computation requirement is not demanding, a MCU can be used, that can save cost and minimize the privacy concerns. The proposed sensing approach can be easily employed into the existing BMS and the real-time cooling demand information can be used in various aspects [63], such as enhancing thermal comfort, saving energy [5,64], controlling outdoor air flow [59] and applying in demand-based control/optimization [3]. Future works will focus on the demand-based control and zone-level indoor environment management to enhance the operating efficiency and thermal comfort of the building cooling systems. 

## Figures and Tables

**Table 1:** Characteristics of different occupancy detection technologies.

Sensing Technology	Type of Sensing	Advantages	Disadvantages	Cost	Commonly Installed? (Yes/No)
temperature or relative humidity sensors	indirect	Opportunities to associate with thermal comfort;	Low accuracy; Low occupancy resolution (often a value of mixing air); Slow response due to the air mixing;	No	Yes
CO_2_	indirect	Associate with indoor air quality;	Easily lead to erroneous measurements due to the diversity in activity level of occupants and envelop infiltrations; Low occupancy resolution (often a value of mixing air); Slow response due to the process of air mixing; Low accuracy and necessity of sensor calibration;	No	Yes
Infrared	direct	High accuracy; Opportunities to associate with thermal comfort;	Unable to detect stationary occupants; Thermal cameras can be expensive; Not commonly installed;	Low (PIR); High (thermal cameras)	No
Wi-Fi or BLE	direct	High accuracy; High covering range;	Require devices to be carried and turning on Wi-Fi or Bluetooth; Signal stability affected by physical obstacles; Occupants outside the physical partition of the room/zone may be miss-counted;	No (Wi-Fi); Medium (BLE)	Yes (Wi-Fi); No (BLE)
RFID	direct	High accuracy; Low cost;	May require tags to be carried (inconvenience on daily activities); Human health concerns of electromagnetic waves; Devices are usually battery-powered and not sustainable for long-term use;	Medium	No
Sound	direct	Moderate accuracy; Opportunities to associate with social behaviors;	Accuracy is easily affected by noise [24,32]; Require multiple sensors;	Low	No
Vision-based detection	direct	High accuracy; High occupancy resolution (i.e., able to obtain occupants’ count or location);	Detection area is limited by camera angle and object occlusion; Privacy or ethical issues; Influence of illumination; May require expensive cameras (e.g., 3D or depth cameras [33]) and computing platforms (e.g., desktop PCs [34]);	No/Low (surveillance cameras); High (advanced cameras)	Yes (surveillance camera); No (advanced cameras)

**Table 2:** General relationship between metabolic rate and density of foreground moving pixels.

Activity	Metabolic Rate—Male Adult ($w/m^{2}$)	Density of Foreground Moving Pixels
Sleeping	40	Very low
Standing, relaxed	70	Low
Office work (reading writing, typing)	55–65	Low
Walking (0.9 m/s)	120	Moderate
Walking (1.8 m/s)	220	High

**Table 3:** Information summary of the university design studio.

Test room Area: 45 m^2^			Maximum Number of Occupants: 30 Persons		
Equipment: One Projector and One PC			Lighting: Eight Luminaries		
Chiller Water Temperature	Measured Supply Water Temperature	8.1–10.2 °C		Normal operation schedule	07:00–23:00 (Weekdays)
	Measured Return Water Temperature	14.5–17.5 °C			07:00–18:00 (Saturdays)

**Table 4:** Summary of data collected.

Source	BMS	Online Weather System	Camera
Information	CO_2_ × 2, Return and supply air temperature	Temperature & Relative humidity	Real-time Streaming Video
No. of information	4	2	1
Connection Type	Internet	Internet	USB
Sensor accuracy	Temperature sensor: Sensirion STS30, ±0.2 °C at a temperature range of 0 °C to 65 °C; CO_2_ sensor: Telaire 7001, ±50 ppm or ±5% of reading up to 5000 PPM	N/A	N/A

**Table 5:** Selected experimental days and weather statistics (in the year of 2016).

**Experimental Days**				
**Month**	**February**	**March**	**April**	**May**
Date	29	1–4, 7–10, 14–18, 28–31	18–22, 25–29	2
**Weather Statistics**				
	**Maximum**	**Minimum**	**Mean**	**Standard Deviation**
Temperature (°C)	30	10	21.24	4.34
Humidity (%)	100	25	79.39	13.99

**Table 6:** Values of the tested DFMP with Metabolic Heat Generation Rate.

Activity Type	Metabolic Heat Generation Rate (w/m^2^)	DFMP (%)
walking—1.8 m/s	220	3.12
walking—1.2 m/s	150	2.83
walking—0.9 m/s	115	2.47
walking about	100	1.83
standing relaxed	70	1.24
reading & writing	55	0.1
sleeping	40	0.028

## Nomenclature

AHU	Air handling unit
ANN	Artificial neural network
BLE	Bluetooth Low Energy
BMS	Building management system
CAV	constant air volume
DFMP	Density of foreground moving pixels
GMM	Gaussian mixture model
HVAC	Heating, ventilation and air conditioning
MCU	micro controller unit
PI	proportional-integral
PIR	Passive infrared
R	correlation coefficient
RFID	Radio-frequency identification
RMSE	root mean squared error
